# The role of *Culex territans* mosquitoes in the transmission of *Batrachochytrium dendrobatidis* to amphibian hosts

**DOI:** 10.1186/s13071-023-05992-x

**Published:** 2023-11-16

**Authors:** Joanna M. Reinhold, Ella Halbert, Megan Roark, Sierra N. Smith, Katherine M. Stroh, Cameron D. Siler, David S. McLeod, Chloé Lahondère

**Affiliations:** 1 Department of Biochemistry, Virginia Polytechnic Institute and State University, Blacksburg, VA 24061 USA; 2 Oberlin College, Oberlin, OH 44074 USA; 3 University of Virginia’s College at Wise, Wise, VA 24293 USA; 4 Sam Noble Oklahoma Museum of Natural History and School of Biological Sciences, The University of Oklahoma, Norman, OK 73072 USA; 5 Murphy Deming College of Health Sciences, Mary Baldwin University, Staunton, VA 24401 USA; 6 The Fralin Life Science, InstituteVirginia Polytechnic Institute and State University, Blacksburg, VA 24061 USA; 7 Center of Emerging, Zoonotic and Arthropod-Borne Pathogens, Virginia Polytechnic Institute and State University, Blacksburg, VA 24061 USA; 8 The Global Change Center, Virginia Polytechnic Institute and State University, Blacksburg, VA 24061 USA; 9 Department of Entomology, Virginia Polytechnic Institute and State University, Blacksburg, VA 24061 USA

**Keywords:** Blood-feeding, Chytrid fungus, Host preference, Mosquito-borne disease, Pathogen transmission

## Abstract

**Background:**

Mosquitoes are the deadliest organisms in the world, killing an estimated 750,000 people per year due to the pathogens they can transmit. Mosquitoes also pose a major threat to other vertebrate animals. *Culex territans* is a mosquito species found in temperate zones worldwide that feeds almost exclusively on amphibians and can transmit parasites; however, little is known about its ability to transmit other pathogens, including fungi. *Batrachochytrium dendrobatidis (Bd) *is a topical pathogenic fungus that spreads through contact. With amphibian populations around the world experiencing mass die-offs and extinctions due to this pathogen, it is critical to study all potential modes of transmission. Because *Cx. territans *mosquitoes are in contact with their hosts for long periods of time while blood-feeding, we hypothesize that they can transmit and pick up *Bd*.

**Methods:**

In this study, we first assessed *Cx. territans *ability to transfer the fungus from an infected surface to a clean one under laboratory conditions. We also conducted a surveillance study of Bd infections in frogs and mosquitoes in the field (Mountain Lake Biological station, VA, USA). In parallel, we determined *Cx. territans *host preference via blood meal analysis of field caught mosquitoes.

**Results:**

We found that this mosquito species can carry the fungus to an uninfected surface, implying that they may have the ability to transmit Bd to their amphibian hosts. We also found that Cx. territans feed primarily on green frogs *(Rana clamitans)* and bullfrogs *(Rana catesbeiana)* and that the prevalence of Bd within the frog population at our field site varied between years.

**Conclusions:**

This study provides critical insights into understanding the role of amphibian-biting mosquitoes in transmitting pathogens, which can be applied to disease ecology of susceptible amphibian populations worldwide.

**Graphical Abstract:**

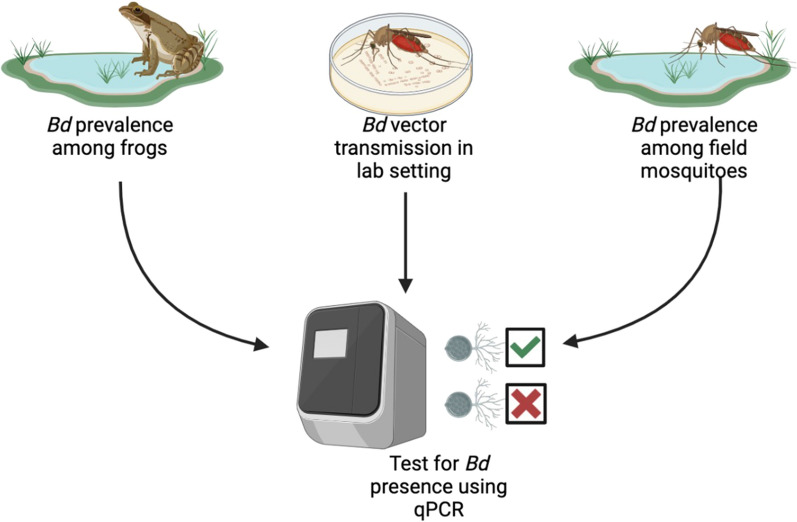

## Background

Mosquitoes are the deadliest animals in the world, spreading many pathogens to their hosts [1, 2]. Although most vectors feed on endothermic hosts such as mammals or birds, some species are known to be ectotherm specialists [3, 4]. A primary example is *Culex territans* Walker 1856, a species of mosquito found in temperate and subtropical zones across the Northern Hemisphere [5–9] that feeds preferentially on amphibians, especially anurans (i.e., frogs and toads) [10–12]. Despite its widespread distribution, little is known about this mosquito. *Culex territans* is a potential vector of pathogens such as giant anuran trypanosomes, hepatozoon parasites [12–14], and viruses [15, 16], but its role in transmitting other pathogens, such as fungi, is unknown. *Batrachochytrium dendrobatidis* (*Bd*) is a topical pathogenic fungus affecting the keratinized tissue in amphibians and is a major contributor to declines in amphibian populations worldwide [17–20]. Chytridiomycosis, the disease caused by *Bd*, affects anuran amphibians more than other amphibian orders, with pathogenic effects varying significantly among species [21, 22]. As an aquatic fungus, *Bd* can be spread environmentally through water or by physical contact with other infected frogs [23]. Fish, crustaceans, and reptiles are known carriers of *Bd* [24–28]; however, the roles that vectors might play in transmitting this deadly fungus remains poorly understood [29, 30]. Toledo et al. [29] found *Bd* DNA present on biting midges (*Corethrella* spp.), showing that arthropod vectors can play a role in the transmission of this fungus. Mosquitoes are known to harbor fungi [31, 32], and *Cx. territans* has a long period of contact time with the skin surface of the frog during blood-feeding (minimum of 30 min to feed to repletion [33]). This amount of time could allow the proboscis and the legs to acquire any fungal spores present on the skin surface of their host. Interestingly, a closely related mosquito species, *Cx. quinquefasciatus*, which feeds on birds and mammals, has been shown to be able to acquire the fungus and transport it to a new surface [30]. Our study tests the ability of *Cx. territans*, the northern frog-biting mosquito, to transfer *Bd* to amphibian hosts, as *Cx. quinquefasciatus* is not known to feed on amphibians.

In the present study, we hypothesized that *Cx. territans* could acquire *Bd* spores and transmit them to a new surface, thus demonstrating the ability to vector this fungal pathogen to anuran hosts through mechanical transmission (i.e., by spreading spores present on the mosquito legs to the skin of their hosts). To test this hypothesis, we first conducted blood meal analyses to determine the primary hosts for this specific population of *Cx. territans*. We then screened field-caught mosquitoes and frogs for the presence of *Bd* at our field site, Mountain Lake Biological Station (MLBS, Pembroke, VA, USA), and carried out a controlled experiment to evaluate the ability of *Cx. territans* to transmit the fungus from one medium surface to another. This work will provide a deeper insight into mosquito-borne disease ecology as well as understanding the potential *Cx. territans* has for vectoring *Bd*, which will enhance our understanding of the spread of this deadly fungus.

## Methods

### Mosquito sample collection

**Fig. 1:**
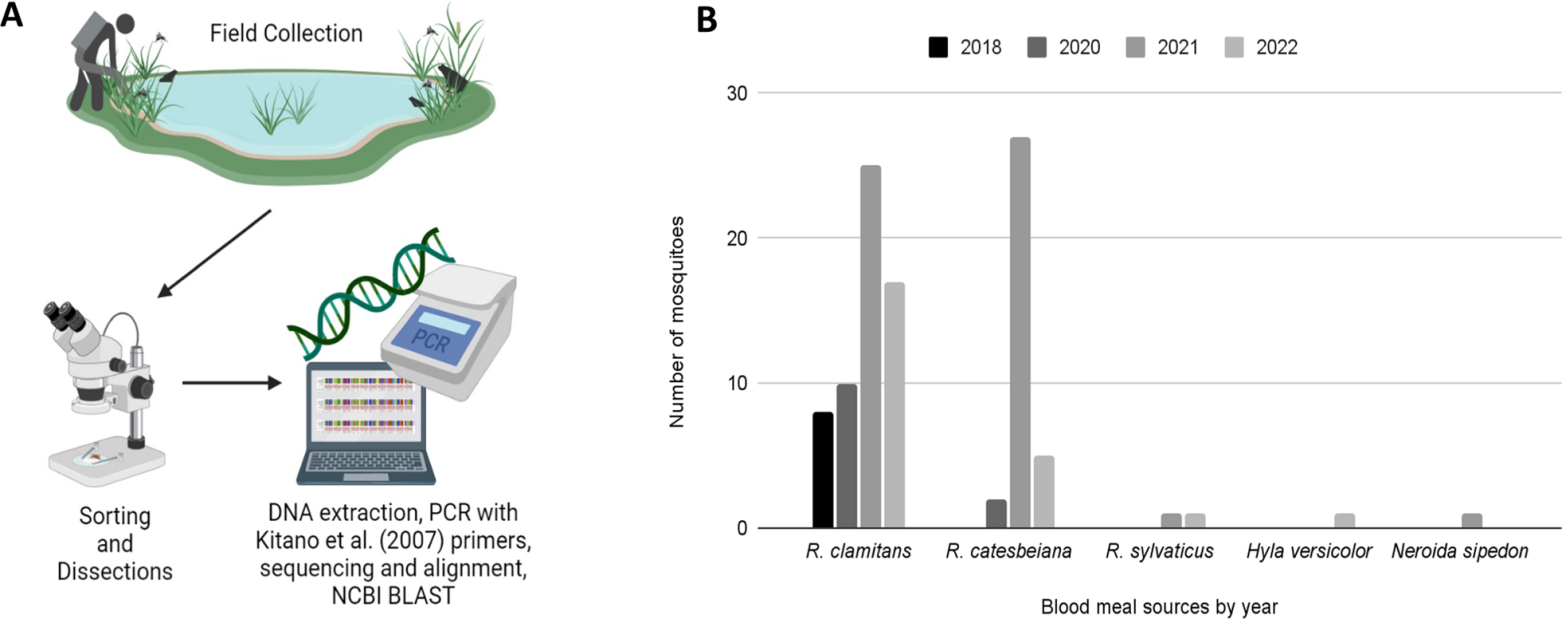
**A** Mosquitoes were collected using a giant bug aspirator around a pond occupied by *R. clamitans* and other anurans. Female *Cx. territans* were sorted out, and blood meal analysis was performed. **B** Blood meal sources of *Cx. territans* mosquitoes by year between 2018 and 2022

Mosquitoes were collected at MLBS (37.375654°–80.522140°, 1160 m above sea level [ASL]) in Giles County, VA, USA. Mosquitoes were collected while at rest around midday from May through September at Sylvatica Pond (37.377079, −80.522245) using a giant bug aspirator (John W. Hock Company, Gainesville, FL, USA) [34]. Following collection, the mosquitoes were brought to the MLBS laboratory, cold-anesthetized, and sorted under a microscope based on species, sex, and reproductive stage [6]. Blood-fed *Cx. territans* females were put in individual 2-ml Eppendorf tubes (Cat #24-283S, Genesee Scientific, Rochester, NY, USA) and were immediately frozen at −80 °C until use for blood meal analysis and pathogen screening for the presence of *Bd* (Fig. 1A), and any bycatch was released.

### Blood meal analysis

Blood-fed mosquitoes collected in the field were thawed, and the abdomens (containing the blood meals) were removed using fine forceps. Abdomens were homogenized using a homogenizer and pestle, and DNA was extracted using Qiagen DNeasy Blood & Tissue Kit (Qiagen, Hilden, Germany), following the manufacturer’s instructions (without the purification step, eluted in 20 µl AE buffer). We amplified the mitochondrial 16S ribosomal RNA (rRNA) gene from each mosquito blood meal via polymerase chain reaction (PCR) using a 50 µl solution containing 25 µl DreamTaq Green PCR Master Mix (2×) (Cat #FERK1081 Thermo Fisher Scientific, Waltham, MA, USA), 21 µl PCR-grade water (CAS: 7732-1-8-5, Sigma Aldrich, St. Louis, MO, USA), 1 µl of forward and reverse primers (L2513: 3′-GCCTGTTTACCAAAAACATCAC-5′; H2714: 3′-CTCCATAGGGTCTTCTCGTCTT-5′) [35] and 2 µl DNA. Amplification was carried out using a thermal cycler (MyCycler, Bio-Rad, Hercules, CA, USA) with 35 cycles of 95 °C for 30 s, 57 °C for 15 s, and 72 °C for 30 s. PCR products were run on a 1% agarose gel containing 0.1% GelRed Nucleic Acid Gel Stain (Biotium, Fremont, CA, USA) for 30 min at 110 V to confirm successful amplification (Bio-Rad), with the amplified product sent to Genewiz (South Plainfield, NJ, USA) for Sanger sequencing. Sequences were aligned using the Qiagen CLC Genomics Workbench (Qiagen, Hilden, Germany) [36], and analyzed using the National Center for Biotechnology Information (NCBI) Basic Local Alignment Search Tool (BLAST). Identification of the source of mosquito blood meals was determined for sequences with a sequence identity of 85% or higher [37] (Fig. 1A).

### Anuran collection and swabbing

For taxonomic stability, we follow the nomenclature of AmphibiaWeb (https://amphibiaweb.org). Anurans (frogs) were collected by hand at three permanent artificial ponds (naturalized for over 50 years) at MLBS: Riopel (37.374552, −80.552109), Sylvatica (37.377079, −80.522245), and Horton (37.378483, −80.522078). The largest of these ponds, Riopel, is approximately 80 × 90× 4 m, whereas the smaller two fluctuate in depth (≤ 1 m) depending on rainfall and measure approximately 30 × 30 m. None of the ponds contain fish. Frog sampling was conducted under Virginia Department of Game and Inland Fisheries collection permit nos. 064750 and 070446 and Virginia Tech Institutional Animal Care and Use Committee (IACUC) protocols (#19-003 and #22-066). Based on the results obtained for the mosquito blood meal analysis, collections were limited to male and female *Rana* (*Aquarana*) *clamitans* and *Rana* (*Aquarana*) *catesbeiana*. Individuals were placed and kept in unused polyethylene bags (25.4 × 40.64 cm 4 Mil Industrial Poly Bags, Uline, Pleasant Prairie, WI, USA) with spring water and tied to prevent escape. Larger frogs were kept in individual terrariums (23.5 × 15.24 × 17.78 cm Kritter Keeper) (Lee’s Aquarium & Pet Products, San Marcos, CA, USA), which were cleaned using a 10% bleach solution between uses to prevent contamination. During handling, nitrile gloves were worn and changed between each individual to prevent potential cross-contamination. Adult frogs were swabbed for *Bd* (described below), measured (snout–vent length), weighed using a spring scale (Pesola, Schindellegi, Switzerland), and marked using the toe clipping method described by Martoff [38] prior to release.

**Fig. 2:**
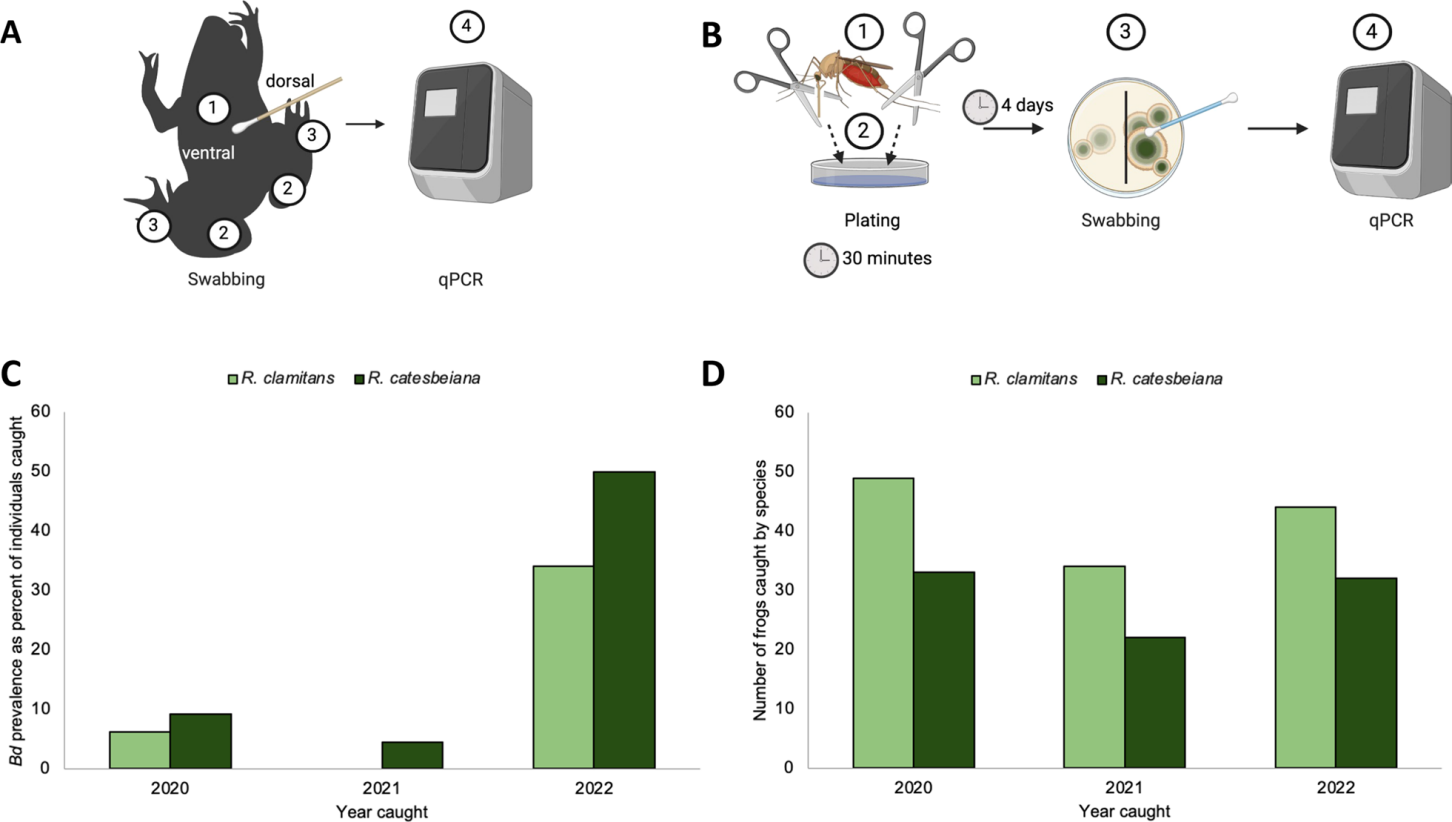
**A** Frogs were swabbed five times each on the (1) abdomen, (2) both medial hind thighs, and (3) between the hind digits of both feet. Swabs were (4) sent out for qPCR to confirm *Bd* presence or absence. **B** The (1) proboscis and legs of field-caught mosquitoes were removed and (2) placed on a sterile agar plate, which was divided to separate the two body parts. After 4 days, the plates were (3) swabbed and (4) sent out for qPCR and analysis. **C** Numbers of frogs caught and swabbed per species between 2020 and 2022. **D** Representation of the numbers of frogs that tested positive for *Bd* from 2020 to 2022

Adult frogs of the two species and sexes were swabbed using rayon-tipped swabs (MW113 series urethral swab, Medical Wire & Equipment Co Ltd, Corsham, UK). The skin of each individual was swabbed five times each on the venter, each medial thigh, and between the digits of each hind leg (Fig. 2A). Swabs were frozen at −80 °C and sent for *Bd* screening at the Genetics Core Facility of the Sam Noble Museum, University of Oklahoma.

### Experimental transmission assays

**Fig. 3:**
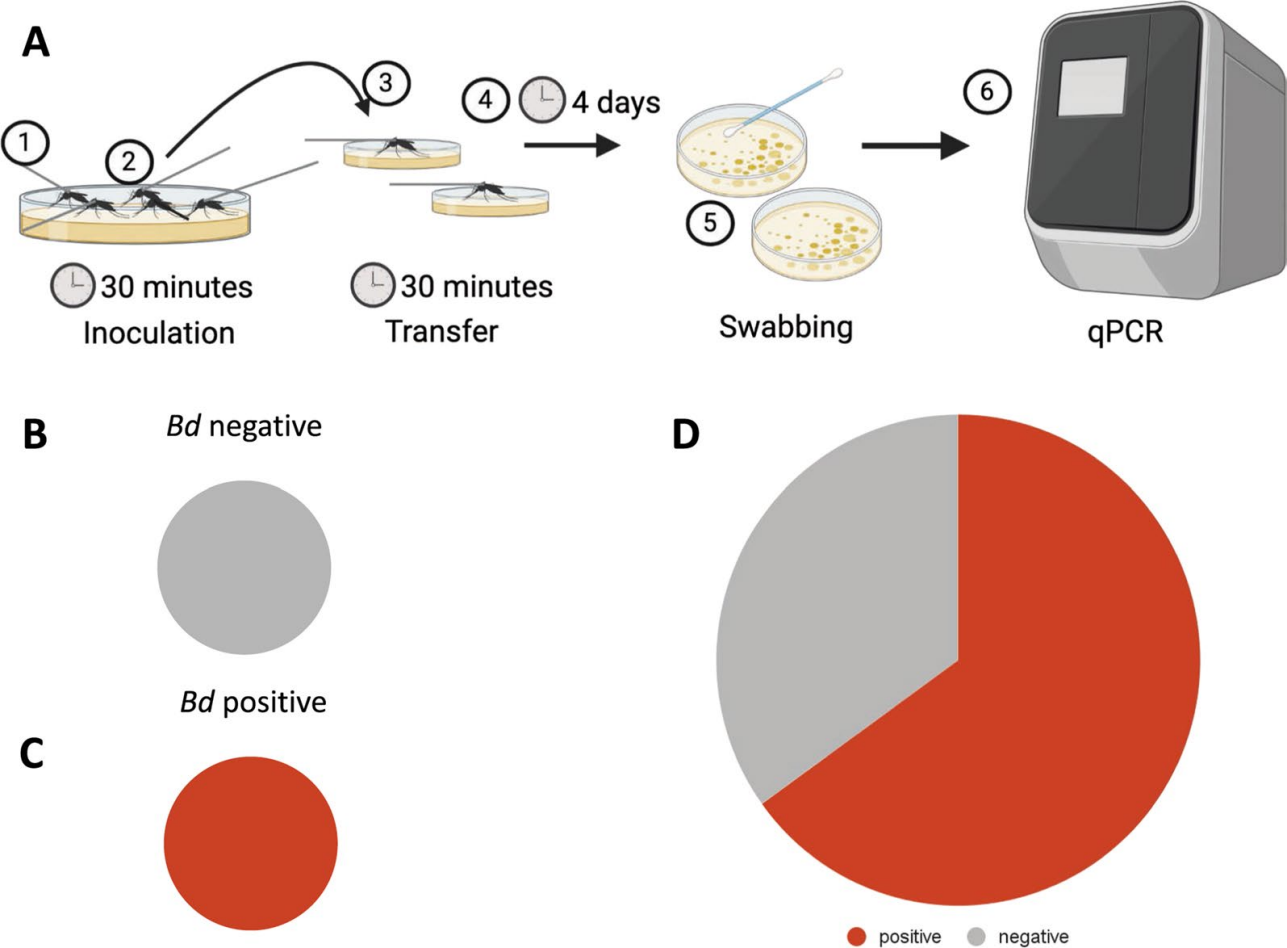
**A** Mosquitoes were (1) tethered to a tungsten rod and (2) placed on a *Bd* plate with legs touching the agar. After 30 min, the mosquitoes were (3) transferred to a new, individual sterile plate with limbs in contact with the agar for another 30 min and (4) let sit for 4 days. After 4 days, the plates were (5) swabbed and stored at −80 °C until they were (6) sent out for qPCR and analysis. **B** Representation of negative controls (*n* = 56). **C** Representation of *Bd*-infected plates to confirm the presence of *Bd* (*n* = 6). **D** The number of plates that were positive for *Bd* 4 days after contact with an inoculated mosquito (*n* = 123)

*Culex territans* mosquitoes were caught as larvae in the ponds at MLBS and reared in the laboratory under conditions as described in Reinhold et al. [39]. Female *Cx. territans* were tested for transmission using a modified experimental setup after Gould et al. [30]. All experiments were conducted in a biosafety cabinet at 25–27 °C using standard sterile practices to maintain the sterility of the plates. *Batrachochytrium dendrobatidis* Global Panzootic Lineage (GPL) strain JEL0423 (Collection of Zoosporic Eufungi at the University of Michigan [CZEUM], Ann Arbor, MI, USA) was plated on sterile 1%T media using CZEUM protocols (10 g Fisher BioReagents tryptone BP1421-500; Fisher Scientific, Waltham, MA, USA) and 10 g bacteriological-grade agar [IBI Scientific CAS #9002-18-0, Dubuque, IA, USA], in 1 L deionized water). Mosquitoes were briefly cold-anesthetized and tethered by the thorax to tungsten rods with ultraviolet (UV)-cured glue (Bondic, Niagara Falls, NY, USA), which held the mosquitoes stationary when exposed to a plate containing *Bd* (cell count: 5.251 × 105 ± 9.727 × 105) (50 × 11 mm petri dishes, #EW-14005-24, Advantech, Taipei City, Taipei, Taiwan). These plates were swabbed to confirm the presence of *Bd* after mosquito exposure. An average of ten mosquitoes (8 < *n* < 12) were held on the *Bd* containing plate for 30 min (*N* = 123, 13 replicates, with some mosquitoes lost during transfer). These mosquitoes were then transferred to new individual sterile 1%T agarose plates for 30 min of contact time to simulate the average time needed for this mosquito species to feed to repletion on a frog host [33]. Plates were incubated at room temperature for 96 h before being swabbed. Swabs were sent to the Genetics Core Facility of the Sam Noble Museum, University of Oklahoma, to confirm the transfer of *Bd*. To control for environmental or potential previous *Bd* exposure (during larval or pupal stage), the same experiment was conducted by exposing 50 mosquitoes (in 5 sets of 10) to sterile plates instead of *Bd*-inoculated plates (*N* = 50). These mosquitoes were also transferred to new individual plates after 30 min and allowed to maintain contact for 30 min. Plates were swabbed after 96 h and sent out for processing as described previously (Fig. 3A).

### Field mosquito *Bd* sampling

A total of 82 field-caught, blood-fed *Cx. territans* mosquitoes [57 and 25 from 2021 and 2022, respectively] were sampled for *Bd*. Sterile 1%T agar plates (50 × 11 mm petri dishes, #EW-14005-24, Advantech, Taipei City, Taipei, Taiwan) were made following CZEUM instructions to simulate the landing onto the surface of an amphibian’s skin. Each plate was divided into 2 sides for the proboscis and legs respectively. The proboscis was clipped using fine-tipped forceps and put on one side of the plate, and the legs from the same mosquito were pulled and placed on the other side of the plate. Forceps were cleaned and dried before removing any parts of the mosquitoes and in between individuals. Plates were incubated at room temperature for 96 h before being swabbed with MW113 swabs. Swab samples were stored at −80 °C until being sent out for processing as described previously (Fig. 2B).

### Screening samples using qPCR

Pathogen screening for *Bd* was conducted at the Genetics Core Facility of the Sam Noble Museum at the University of Oklahoma. Swabs from both agar plates and frogs were first extracted using the PrepMan Ultra Sample Preparation Reagent protocol [40] (Applied Biosystems, Thermo Fisher Scientific). Quantitative PCR (qPCR) techniques were used to determine the presence/absence of *Bd* genetic material and to estimate the number of gene copies per sample, or *Bd* load, using QuantStudio software v3 (Applied Biosystems, Thermo Fisher Scientific). The *Bd* assay primers target the internal transcribed spacer (ITS-1) rRNA gene (forward primer: ITS1-3 Chytr; reverse primer: 5.8S) from Boyle et al. [41]. All samples were run in triplicate, with positive controls of known *Bd* gene copies (gBlock DNA quantities 1e1–1e4) and a negative control (molecular grade sterile water). Samples were considered positive for *Bd* (*Bd*+) if amplification occurred in at least two of the three wells and if the mean *Bd* gene copy number was greater than 1.0, with samples rerun as needed [42, 43].

## Results

### Blood meal analysis

A total of 538 mosquitoes were collected from Sylvatica Pond at MLBS. Among them, 98 were blood-fed *Cx. territans*. We identified a total of five host species, including *R. clamitans* (61%) (green frog), *R. catesbeiana* (35%) (American bullfrog), *Rana sylvatica* (2%) (wood frog), *H. versicolor* (1%) (gray treefrog), and *Nerodia sipedon* (1%) (northern water snake). We noted that *Cx. territans* preferentially fed on *R. clamitans* (61%) and *R. catesbeiana* (35%) (Fig. 1B).

### Frog collection and swabbing

A total of 127 *R. clamitans* and 87 *R. catesbeiana* were caught and swabbed between 2020 and 2022 (Fig. 2A, C). Among the individuals swabbed, 17.8% were confirmed as *Bd*+. The prevalence of *Bd* infection among the surveyed populations varied between years and species: 9.1% prevalence in *R. catesbeiana* and 6.1% in *R. clamitans* during 2020 dropped to 4.5% and 0% in *R. catesbeiana* and *R. clamitans*, respectively, in 2021. During 2022 we found 38% and 50% prevalence among *R. catesbeiana* and *R. clamitans*, respectively (Fig. 2D). None of the 82 field-caught mosquitoes screened for *Bd* were positive.

### Transmission assays

Of the 123 experimental mosquitoes tested, 80 were positive for *Bd* (65%) (cell counts 5.251 × 10^5^ ± 9.727 × 10^5^; gene copies 1.94499 × 10^5^ ± 3.37174 × 10^5^) (Fig. 3D). None of the five initial control plates or the 50 test controls were positive for *Bd*, showing no evidence of prior exposure to the fungus (Fig. 3B). All *Bd*-inoculated plates contained *Bd*, confirming the experimental mosquitoes were exposed to the fungus (Fig. 3C).

## Discussion

This study was conducted to assess the prevalence of the pathogenic fungus at our field site, MLBS, and to ascertain whether the amphibian-biting mosquito, *Cx. territans*, is capable of vector transmission of *Bd* to anurans. We determined the primary hosts of *Cx. territans* and tested two focal hypotheses: (1) Pathogen screening efforts would detect *Bd* on *Cx. territans* and their anuran blood meal hosts in the field, and (2) *Cx. territans* is capable of *Bd* transmission from one surface to another in a laboratory setting.

Although *Cx. territans* has been known to feed on a wide variety of hosts [10–12], our results show that the mosquito population at MLBS exhibits a strong preference for *R. clamitans* and *R. catesbeiana* (Fig. 1). Both anuran species are commonly observed around the MLBS ponds. A plethora of other potential hosts have been observed at the MLBS ponds, including *R. sylvatica*, *Rana palustris* (pickerel frog) *H. versicolor*, and *Pseudacris crucifer* (spring peeper). Additional potential hosts present at MLBS include *N. sipedon* (northern water snake), *Crotalus adamanteus* (eastern diamondback rattlesnake), and numerous salamander species (in particular *Notophthalmus viridescens*, and several *Desmognathus* and *Plethodon* spp.) [44]. The strong host preference for *R. clamitans* and *R. catesbeiana* exhibited by *Cx. territans* led us to focus our *Bd* screening on these two species.

Overall, we found that the current prevalence of *Bd* is relatively low among the *R. clamitans* and *R. catesbeiana* populations at MLBS. Previous studies at MLBS found several species of amphibians carrying *Bd*. Rothermel et al. [45] and Wimsatt et al. [46] found *Bd* present in all *Notophthalmus viridescens* (red-spotted newt) individuals tested at MLBS, which is present in all three ponds at the station where *R. clamitans* and *R. catesbeiana* are found. While these studies only tested one species, they demonstrate that *Bd* has been present in the MLBS ponds for more than a decade. Hughey et al. [47] also reported *Bd*-infected individuals of *N. viridescens*, *P. crucifer*, and *R. catesbeiana*, and found lower rates of infection among *R. catesbeiana* than the other two species. None of these studies associated *Bd* infections at MLBS with die-offs or significant population declines. Collectively, the present and previous studies show that *Bd* is established at MLBS. Our results also suggest that infection prevalence and detectability vary from year to year (Fig. 2D). This kind of variability is not uncommon in areas where *Bd* is considered endemic [47–52] and may be part of natural long-term oscillations described by Talley et al. [49]. Several factors could be playing a role in these oscillations. The presence of carriers in these areas, including (but not limited to) *N. viridescens*, *R. catesbeiana*, and crayfish (e.g., *Cambarus* spp.) may be acting as fungal reservoirs and may partially account for the yearly fluctuations we detected in anuran populations [24, 26, 27, 45–47, 53–55]. Environmental factors could also affect *Bd* prevalence, with colder and drier years potentially causing a shift in *Bd* prevalence [20, 23, 49, 51, 56, 57]. Despite the sudden increase in *Bd* in 2022, no mass die-offs of amphibians were observed at the time, which suggests that *Bd* is not an immediate concern for the amphibians at the site, but future observations and surveillance will provide more insight.

Our experimental transmission test confirmed that *Cx. territans* can transmit *Bd* from one surface to another (Fig. 3D). This suggests that *Cx. territans* can transfer the fungus to a new host after landing and feeding on an infected host. Ecologically, *Cx. territans* is a good candidate to vector a topical fungus, given that it feeds primarily on frogs and is very slow to feed to repletion [10–12, 33]. If the mosquito does not have the opportunity to feed to repletion (i.e., frog jumps into the water), they may seek a new host to complete their meal, allowing them to transmit *Bd* zoospores between hosts. They may also transmit the fungus between gonotrophic cycles, though further testing is needed to determine the maximum time the mosquito can carry the fungus. Because 35% of the mosquitoes did not transmit *Bd* to a new plate, there may be a threshold concentration necessary for transmission. The large range in cell counts on each inoculated *Bd* plate could also have contributed to the variability in individual mosquitoes’ ability to transmit zoospores between plates. Another source of variability could be the uneven nature of the way the *Bd* fungus grows. All mosquitoes were placed on visible fungus, but it was difficult to determine which plated area was actively releasing zoospores. This may provide a more ecologically relevant way of testing the ability of a mosquito to transmit *Bd* experimentally than has been previously shown in the literature [30], since the landing area of the mosquito on a frog may not be releasing zoospores. Because more than two-thirds of the mosquitoes transmitted *Bd* to uninfected surfaces, we conclude that *Cx. territans* can carry spores on their appendages and could thus be a competent vector of *Bd* in the field.

We hypothesized that we would find *Bd* on the mosquitoes caught in the field, but all field-caught mosquitoes were negative for *Bd*. These results are not unexpected, considering the low prevalence of the fungus in the frog population in 2020 and 2021 and the small mosquito sample size for 2022. However, based on the ability of other arthropod vectors to act as carriers of *Bd* in the wild [29] and the ability of *Cx. territans* to transmit the fungus in a laboratory setting, we would expect to find *Bd*-carrying mosquitoes in a larger sample or among samples from a more highly infected population of frogs. Because this mosquito species lives in close association with frogs throughout the Northern Hemisphere and given that *Bd* is also prevalent in these regions [5–10], transmission in areas of high *Bd* prevalence is likely. The lack of *Bd* found on field mosquitoes may also be due to the location of *Bd* on frogs. *Rana clamitans* and *R. catesbeiana* were often observed resting partially submerged in water, as noted in previous studies [38, 58]. This position results in their ventral surfaces being more likely to be exposed to the water-borne fungus, and consequently, unless the fungus has spread extensively, a mosquito feeding on the dorsum of a frog’s body may not make contact with the fungus. Mosquitoes were observed in the field clustering on the frogs’ back legs and lower back, possibly to avoid detection and antiparasitic behavior (e.g., grooming) from the host as seen in other mosquito species [59–61]. We also observed mosquitoes on the ventral surfaces of the body and on the toes when those areas were exposed. *Culex territans* is known to feed on more terrestrial amphibians and reptiles [10, 11]. It would therefore be beneficial for future studies to collect additional *Cx. territans* mosquitoes and test for field transmission, especially in areas where *Bd* prevalence is high.

## Conclusion

In this study, we determined the host preference of *Cx. territans*, the prevalence of *Bd* at the MLBS field site, the ability of *Cx. territans* to transmit *Bd* in a laboratory setting, and *Bd* prevalence among blood-fed mosquitoes in the field. We found that, at MLBS, *Cx. territans* feeds preferentially on *R. clamitans* and *R. catesbeiana*. We observed varying *Bd* prevalence over the course of 3 years at MLBS within *R. clamitans* and *R. catesbeiana* populations. Most importantly, we found that *Cx. territans* can transmit the fungus to a clean surface. We did not, however, find mosquitoes in the field with *Bd* present on their proboscis or legs. Overall, the results of this study add to the growing understanding of *Bd* epidemiology and disease ecology both on a local and global scale. Future research will help determine whether mosquitoes in the field can carry the fungus and transmit it to a new host as well as the length of time the fungus can stay on the mosquito.

## Data Availability

The data supporting the findings of this study are available within the article.

## Abbreviations

*Bd*: *Batrachochytrium dendrobatidis*
*Cx*: *Culex*
*R*: *Rana*
MLBS: Mountain Lake Biological Station
*H*: *Hyla*
*N*: *Notophthalmus*
PCR: Polymerase chain reaction
qPCR: Quantitative polymerase chain reaction
rRNA: Ribosomal RNA
